# A Comparison of Environmental Impact of Various Silicas Using a Green Chemistry Evaluator

**DOI:** 10.1021/acssuschemeng.2c00519

**Published:** 2022-04-12

**Authors:** Carlos Brambila, Peter Boyd, Amber Keegan, Pankaj Sharma, Caleb Vetter, Ettigounder Ponnusamy, Siddharth V. Patwardhan

**Affiliations:** † Green Nanomaterials Research Group, Department of Chemical and Biological Engineering, University of Sheffield, Mappin Street, Sheffield S1 3JD, United Kingdom; ‡ Sigma-Aldrich Chemicals Pvt. Ltd. (Merck Group), Tower 2, Electronic City, Bangalore 560100, India; § MilliporeSigma, 545 South Ewing, St. Louis, Missouri 63103, United States

**Keywords:** DOZN, sustainability, bioinspired silica, green nanomaterials, energy, solvents

## Abstract

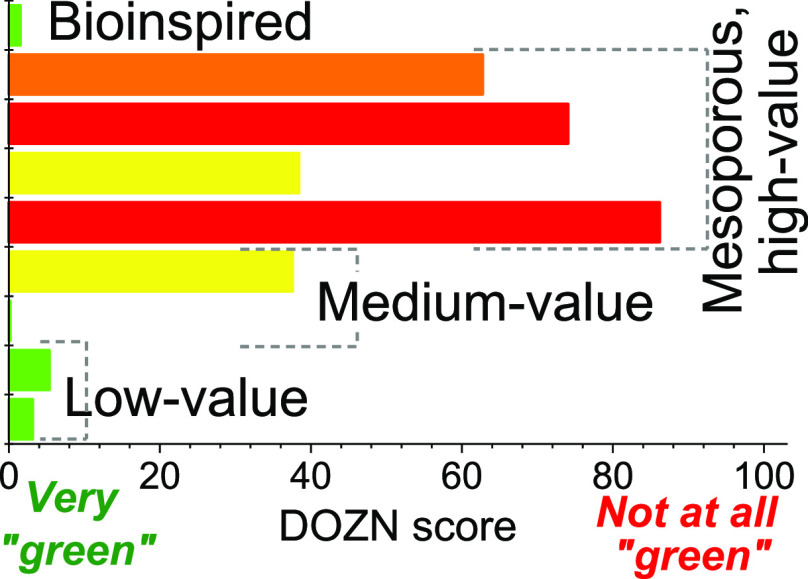

To answer questions surrounding the sustainability of silica production, MilliporeSigma’s DOZN 2.0 Green Chemistry Evaluator was employed as it provides quantitative values based on the 12 principles of Green Chemistry. As a first study using DOZN 2.0 to evaluate the greenness of nanomaterials, a range of silica types were considered and their greenness scores compared. These included low- and high-value silicas, both commercial and emerging, such as precipitated, gel, fumed, colloidal, mesoporous, and bioinspired silicas. When surveying these different types of silicas, it became clear that while low value silicas have excellent greenness scores, high-value silicas perform poorly on this scale. This highlighted the tension between high-value silicas that are desired for emerging markets and the sustainability of their synthesis. The calculations were able to quantify the issues pertaining to the energy-intensive reactions and subsequent removal of soft templates for the sol–gel processes. The importance of avoiding problematic solvents during processes and particularly releasing them as waste was identified. The calculations were also able to compare the amount of waste generated as well as their hazardous nature. The effects of synthesis conditions on greenness scores were also investigated in order to better understand the relationship between the production process and their sustainability.

## Abstract 2

A first study comparing the sustainability of a range of silicas, highlighting areas of concern and identifying potential improvements is presented.

## Introduction

Silica is a core constituent of a variety of products, spanning from rubber^1−3^ to high-precision drug delivery systems.^4−6^ This variety of applications is enabled by the physical and chemical variations that silica can produce under different synthesis and processing conditions. The development of technologies across health and other high-value industries continues to increase the need for silica products with highly controlled and intricate structures and functionalities.^7,8^ The steps in silica synthesis determine the cost, application, and impact of numerous established and emerging products. For that reason, silica technology is an impactful branch of knowledge where every development can improve the capabilities of multiple industries, their environmental effect, and their influence upon our quality of life.

Nanostructured silicas continue to gain particular interest for high-value applications over their bulk counterparts.^9,10^ These materials can be engineered to take advantage of the chemical and physical phenomena that occur on the nanoscale. As such, silica nanomaterials offer a diverse range of properties, from the mesoporous silicas featuring unique porous structures and high surface areas^11,12^ to the amorphous colloidal silicas with tunable optical properties.^13,14^ Mesoporous silicas have proven especially relevant for high-value emerging technologies like catalysis, separations, and drug delivery systems. Adding to their high surface area, uniform pore sizes and pore volumes, mesoporous silicas also benefit from facile functionalization capabilities.^15,16^

Given the relevance of the industries and applications mentioned above, it would be reasonable to expect nanostructured silicas to be found in numerous end-user products and for their manufacture on a large scale to be an established industry. Unfortunately, even the well-known varieties like MCM-41 are difficult to secure in quantities larger than a few hundred grams, and their extremely high production costs impede any industrial scale application or even pilot-plant testing. These issues are likely to be associated with the conditions needed to synthesize mesoporous silicas (e.g., reaction times of hours to days, extremes of pH, and high temperatures), but this has not been quantified yet.

An interesting contrast can be found when comparing the harsh synthesis conditions of mesoporous silicas and the natural occurrence of complex porous structures in biosilica,^17,18^ which takes place under mild conditions such as room temperature, ambient pressure, and aqueous media. Bioinspired synthesis seeks to replicate these highly efficient biological mechanisms by designing synthetic molecules that can enable silica synthesis under mild conditions while allowing control of the structural and functional properties of nanostructured products.^19−21^ This bioinspired approach has also proven promising for other industrially desirable materials including titania,^22^ magnetite,^23^ and zinc oxide nanoparticles.^24^ Bioinspired synthesis has been shown to produce a pure silica product of tunable nanostructure at room temperature.^25^ When considering the functionalization capabilities of bioinspired silica (BIS) and their improved ability to encapsulate drugs and biomolecules, their commercial desirability becomes even more apparent.^26,27^ Nonetheless, some aspects of sustainability can be more complex and entail a variety of factors, which raises the need for a reliable method to quantify the sustainability of the BIS route.

Traditionally, sustainability studies have focused mainly on analyzing the toxicity of a product.^28^ This, while vastly important, is not a useful approach when comparing various processes that produce similar products, as is the case with silica synthesis. Furthermore, if a manufacturing operation results in toxic waste and byproducts, the environmental impact of these should be contemplated as much as the toxicity of the main product. Such limitations exemplify the need for a holistic sustainability evaluation that comprehends all variables that can contribute to the environmental impact of an industrial process.

Several sustainability frameworks have been developed seeking to assess, albeit sometimes qualitatively, the greenness or environmental impact of any specific product. Notable advances include the NSF/GCI/ANSI 355–2011 industrial standard,^29^ which provides a standardized methodology for comparison of chemicals, which improves the transparency of industrial production. However, the scope of this standard does not extend to account for the end-of-life stages of the product. A more promising alternative is the iSUSTAIN Green Chemistry Index developed by Beyond Benign, Cytec Industries, and Sopheon, which focuses on gate-to-gate assessment of health, safety, product use and disposal.^30^ However, by assigning values within the same scale to all results, the user can be misled to think that all factors have a similar impact on the sustainability of the product. These proprietary formulas and ambiguous results can also make this green chemistry metric (GCM) significantly lacking in transparency.

Several major industries have developed their own GCMs for accountability and control throughout their supply chain. A major example can be found in the Selection Guidelines developed by GlaxoSmithKline, which generally use global-warming potential and process mass intensity (PMI) to evaluate the environmental impact of new pharmaceutical compounds.^31^ Other proprietary GCMs developed by the pharmaceutical industry include the Solvent Selection Guide by GSK and Merck.^32^ This metric considers waste prevention, yield analysis, and operational safety for a wide selection of solvents. Other noteworthy efforts include their FLASC (Fast Lifecycle Assessment of Synthetic Chemistry) tool,^33^ which expands on the global warming potential and waste production of products and processes but omits considerations such as accident prevention or atom economy.

While the examples above and other notable GCM frameworks^34−36^ can certainly be used to improve on the sustainability of new and existing processes, none of them target all of the three major goals of green chemistry: minimizing the use and production of hazardous substances, reducing waste, and lowering the demand of nonrenewable resources. These three priorities are comprehensively fulfilled by the 12 principles of Green Chemistry, as developed by Anastas and Warner.^37^ This conceptual framework contemplates both health and environmental risks, as well as resource efficiency from a lifecycle perspective, ranging from raw materials extraction to end-of-life bioaccumulation. The reliability and breadth of application of this framework has led it to be adopted by all major chemical societies.

**Figure 1:**
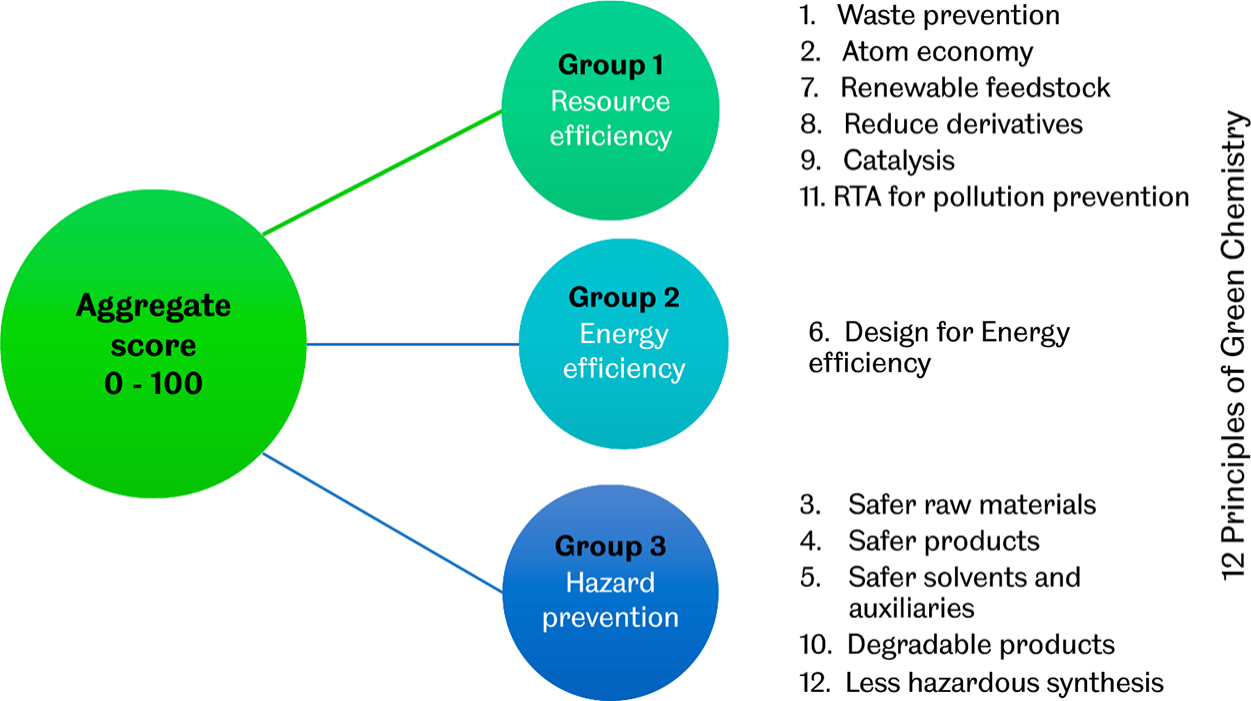
Metric hierarchy used by DOZN 2.0 Green Chemistry Evaluator. The right-hand side shows the 12 principles of Green Chemistry.

A notable limitation to the applicability of the 12 principles lies in its conceptual or qualitative nature. Seeking to overcome these limitations, the DOZN 2.0 Green Chemistry Evaluator was developed as a unique GCM founded upon the 12 principles (Figure 1).^38^ By incorporating standardized calculations into the application of the 12 principles framework, DOZN 2.0 has been able to provide users across different disciplines with reliable sustainability measurements, which have enabled the comparative assessment of greener alternatives for chemistry- and biology-based products. Further details on the description of this tool and the equations constituting the DOZN 2.0 algorithm can be found in the cited literature.^38,39^

Given the flexibility of DOZN 2.0 and its comprehensive consideration of process conditions and lifecycle aspects, it is an ideal method for exploring the major sustainability questions surrounding silica production. Namely, what is the environmental impact of established silicas and, is bioinspired synthesis *quantifiably* greener enough to unlock high-value silica manufacturing? In the present work, we report the greenness assessment of a variety of silica production methods, which have been selected as representative of the most widespread industrial and experimental methods for low- and high-value silicas.

## Methods

A survey of the literature has been conducted to establish representative synthesis methods for the selected materials. Whenever possible, parameters such as reaction times and temperatures have been varied within the ranges reported in the literature. The parameters to be assessed are used as inputs for the DOZN 2.0 algorithm, which outputs comparable numeric results based on its hierarchy of metrics: 12 principle scores, three group scores, and an overall score. These 16 scores for each case have been used to compare arrays of reaction conditions and for comparing across different types or grades of silicas. All cases have been scaled based on 1 g of product to simplify the comparisons. Full details on the calculations behind the DOZN 2.0 scores have been previously published.^38^

### Materials Selection and Boundaries

Table 1 shows a summary of the materials selected for greenness assessment. First, three major industrial products have been chosen to represent bulk manufacturing of low to medium value products: precipitated silica, fumed silica, and silica gel. Stöber synthesis of monodisperse nanospheres is also included as it is a widespread nanomaterial synthesis route. Four mesoporous silica materials were selected based on their popularity as prospective drug carriers and molecular sieves: MCM-41, SBA-15, HMS, and COK12. Finally, amine-assisted bioinspired silica is included as a promising alternative route to high-value silica.

**Table 1:** Summary of Selected Materials Highlighting Their Major Applications and Their Synthesis Process

silica type	applications	synthesis process
precipitated silica	low value: rubber fillers such as tires, free-flow agent	precipitation^40^
silica gel (xerogel)	low value: desiccant, toothpaste, coatings	precipitation or sol–gel^40^
fumed silica	low to medium value: reinforcing fillers, thickening agents, dispersants, excipients	pyrolysis^40^
Stöber nanospheres	low to medium: research materials, potential for biosensing^41^	sol–gel^42^
mesoporous MCM-41	high value: catalytic cracking,^43^ drug delivery,^44^ adsorption^45^	soft-templated sol–gel^46^
mesoporous SBA-15	high value: catalysis,^47^ adsorption, delivery of particularly insoluble drugs^48−50^	soft-templated (pluronic) sol–gel^51^
hexagonal mesoporous silica (HMS)	high value: drug delivery,^52^ adsorption,^45^ catalysis^7,53^	soft-templated (amine) sol–gel^54^
mesoporous COK12	high value: catalysis,^55^ potential for drug delivery and adsorption^56^	soft-templated sol–gel^57^
bioinspired silica	high value: catalysis,^58^ adsorption,^59^ and drug delivery^27^	amine-assisted sol–gel^60^

**Figure 2:**
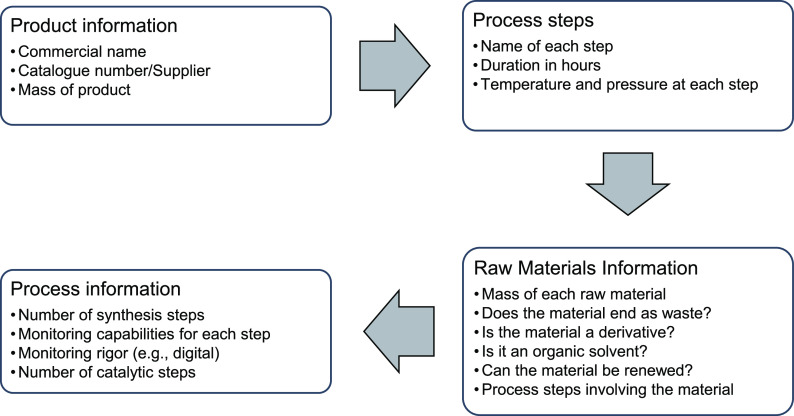
Typical inputs for DOZN 2.0 and the workflow.

Figure 2 shows a typical example of the main inputs required by the DOZN 2.0 tool algorithm. Equivalent variables have been considered for each of the selected materials based on a survey of the literature. Given the relevance and popularity of mesoporous silicas, modifications of their synthetic routes can be found throughout the literature, such as Stöber-based mesoporous silicas^61^ and aerogels;^62^ however, analysis of such modifications is excluded, as the present work is not intended as a comprehensive study of these methods. Instead, the parameter boundaries have been chosen as *representative* of each synthesis route. Moreover, the variables selected for the study have been chosen to represent most commonly studied variables. The typical inputs for DOZN 2.0 are shown in Figure 2, while the synthesis details are given in Table 2 (also see Table S1 with full details of the parameters input).

**Table 2:** A Range of Parameters Considered for Various Silicas

material	parameter	boundaries
mesoporous MCM-41	synthesis time	10–144 h
	synthesis temperature	40–100 °C
mesoporous SBA-15	synthesis time	10–44 h
	synthesis temperature	40–120 °C
mesoporous HMS	purification method	calcination for 4 h at 630 °C or ethanol reflux for 3 h at 45 °C
precipitated silica	synthesis temperature	40–80 °C
silica gel	synthesis time	3–5 h
	synthesis temperature	35–80 °C
	sizing temperature	20–60 °C
fumed silica	deacidification time	5–10 min
	deacidification temperature	200–500 °C
mesoporous COK-12	synthesis temperature	20–90 °C
Stöber nanoparticles	synthesis time	12–24 h
bioinspired silica	purification method	calcination for 6 h at 550 °C or rapid acid elution at room temperature

As shown in Figure 2, the input parameters used for the DOZN 2.0 score calculations consider numerous aspects of the raw materials, the product, and the process. Therefore, it is possible to obtain several sets of greenness scores for the same product when adjusting the input values to account for variations in the process. In line with the cited literature, each synthesis process has been tested with different parameters as shown in Table 2. These parameter variations serve multiple purposes. First, they allowed us to test the reliability of the DOZN 2.0 tool to reflect significant operational changes onto the 12 principle scores. Having established this reliability, DOZN 2.0 could then be used to identify the operational conditions contributing most strongly to the environmental impact of each silica process. This valuable knowledge is made possible by the transparency of the DOZN 2.0 algorithm, which is unique in allowing the user to easily understand how every stage of a chemical process affects the sustainability metrics.

## Results

### A Comparison of Various Silicas

Given that the synthesis procedures for some silicas have similar steps, the descriptions below have been grouped to provide clear comparisons. The comparisons are based on the 12 principle scores, three group scores, or an overall (or aggregate) score. The lower the score, the greener the synthesis is.

**Figure 3:**
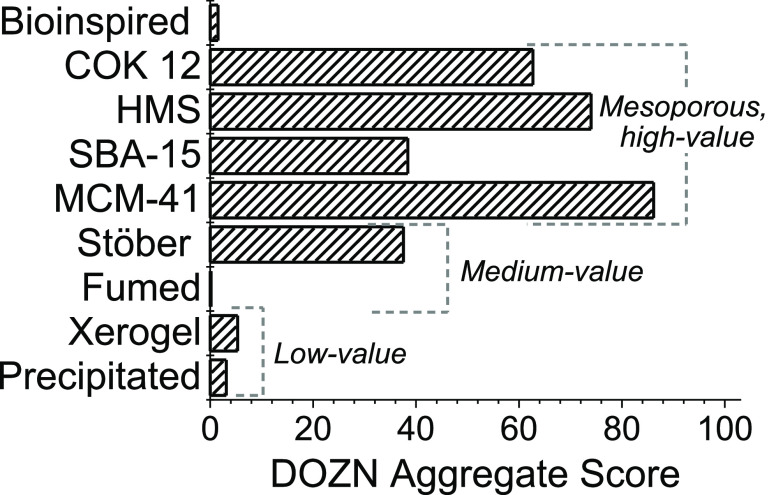
Comparison of overall scores for selected silicas calculated using DOZN 2.0.

Figure 3 shows the aggregate scores for all of the silicas compared in this study. For the overall score, it varies between zero (the most or ideally green) and 100 (the worst or least green). These scores were calculated using all 12 principles of green chemistry. In this section, the overall scores for different types of silicas are compared while further details about the principle and group scores are discussed in subsequent sections. The scores for industrially manufactured bulk silicas are all around 5 or below, which represents a highly optimized and green process. This is consistent with the fact that these processes have been engineered for maximum efficiency and cost reduction.

**Figure 4:**
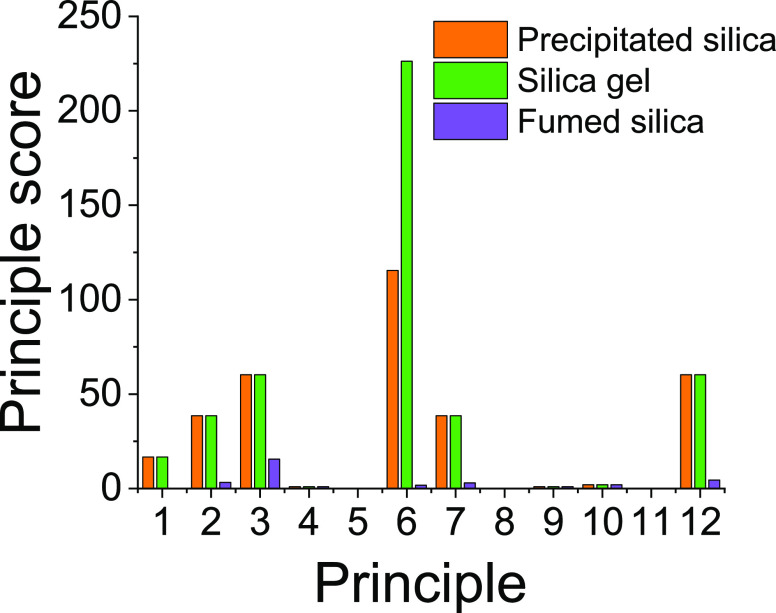
Comparison of major industrial silicas showing the 12 principle scores. The principle numbers correspond to their conventional allocation, as shown in Figure 1 (right-hand side).

The large volume manufacturing of industrial silica resulted in consistently low scores throughout all of the 12 principles, as shown in Figure 4. The effect of industrial engineering and process optimization can be observed in the high resource efficiency as evidenced by principles 1 and 2, waste prevention and atom economy. This comparison of industrially manufactured silicas shows significant variation in the scores for principle 6, i.e., design for energy efficiency (Figure 4). It can be appreciated that the time of the reaction can have as much impact over the score as its temperature. It is for this reason that the principle 6 scores for precipitated silica and silica gel are much higher despite operating at low temperatures (<80 °C) than that of fumed silica, which is pyrolyzed at temperatures of several thousand degrees Celsius but with a dwell time of only seconds.^40^ This in an important observation, suggesting that simply reducing synthesis temperature does not guarantee a greener synthesis. Another interesting learning from the comparison of industrial silicas is seen in principle 12, where again for pyrolyzed silica scores were low. This can be explained by the difference in raw materials. The score for principle 12 is calculated using a raw material’s *P* score, which relates to its Globally Harmonized System’s (GHS) hazard classification category.^38^ In the case at hand, sodium metasilicate or tetraethyl orthosilicate used for precipitated silica or silica gel are categorized as more hazardous than silicon tetrachloride used for fumed silica, which presents lower hazards.^63^

The original Stöber synthesis, , as published in 1968, was also evaluated.^42^ This synthesis route has been repeated and modified many times. Nonetheless, they generally consist of slow reactions using tetraethyl orthosilicate (TEOS) and a high amount of ethanol at an elavated temperature. The Stöber process had a high score, as the least green of all selected silica syntheses, as can be seen in Figure 3. The poor greenness scores of the Stöber process can generally be attributed to the high amounts of ammonia and ethanol required to produce a gram of silica. Also, the energy required to maintain a constant temperature of 60 °C for 24 h resulted in a significantly high score for energy efficiency. Finally, the use of ethanol, ammonia, and tetraethyl orthosilicate was detrimental to the scores relating to safer chemistry for accident prevention. Overall, the Stöber process serves as a good example of a conventional lab-scale process that is not designed for efficiency or sustainability.

The scores for mesoporous silicas varied with the type of silica, mainly due to the differences in energy efficiency of their synthesis. The processes involving slow formation reactions and calcination at high temperatures, such as COK-12 and MCM-41, scored particularly poorly. Moreover, the synthesis of mesoporous silica often relies on highly toxic silica sources, mainly tetraethyl orthosilicate (TEOS), as well as significant amounts of ethanol and other solvents handled at boiling temperatures. These substances not only present significant risks to health and safety but also increase the capital expenditure by requiring specialized equipment for their handling and storage.

The high scores for some of the mesoporous and Stöber silica can explain the difficulties in taking these processes from the lab into the market. Finally, bioinspired silica presented the only score of an experimental procedure that can compete with the industrial silicas. This is likely due to the room temperature nature of the synthesis, as well as the use of room temperature acid elution for its purification instead of calcination or solvent reflux.^60^ To better understand the overall scores for all silicas, an analysis of the contributions for various green chemistry principles to these scores is discussed in the next section.

### Comparison of Silica Syntheses Based on the Group Scores

#### Group 1. Resource Efficiency

**Figure 5:**
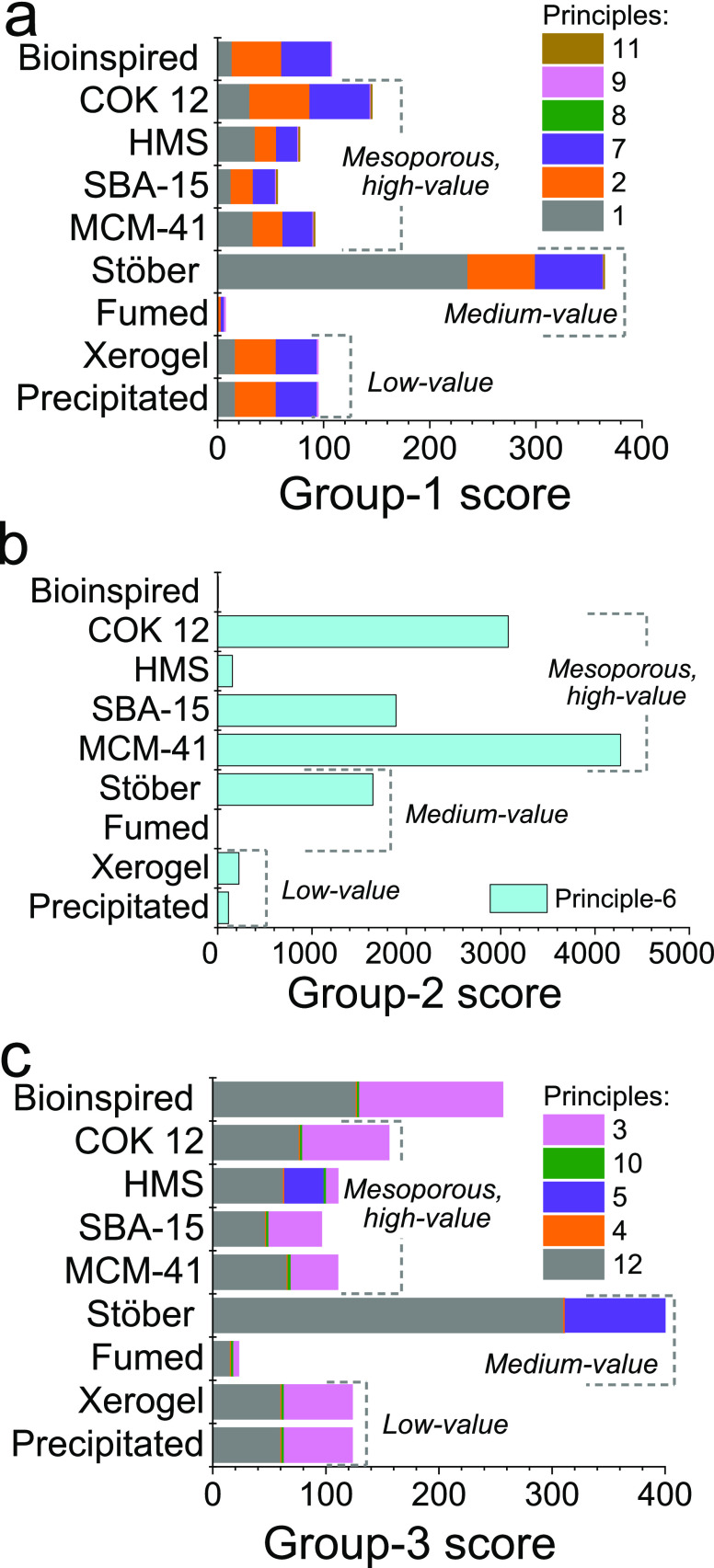
Group scores for selected silicas showcasing the three major aspects of improved processes and products, calculated using DOZN 2.0: (a) group 1, resource efficiency; (b) group 2, energy efficiency; and (c) group 3, hazard prevention. The scores are composed of individual principle scores as denoted by the different color bars (the principle numbers correspond to Figure 1).

This group consists of six principles of green chemistry. Figure 5a shows the group 1 scores for each silica type considered herein and how each principle contributed to it. Given the similarities to the mechanisms driving silica precipitation and polymerization, the scores were generally similar. Significant exceptions were found in the Stöber process and pyrolyzed silica. Mainly, the high production of ethanolic waste from the Stöber synthesis resulted in a comparatively high environmental cost per gram of product (principle 1). On the other hand, the high yields and rapid reaction times of pyrolyzed silica were the main reasons behind the low environmental impact as calculated.

Bioinspired silica ranked seventh out of the nine silicas for resource efficiency. The main contributors to this ranking were the atom economy and use of renewable feedstock. These were both skewed highly due to the yield (discussed in the next section). Apart from this, bioinspired silica scored well for greenness, achieving the third rank for waste prevention (principle 1) and joint top ranks for reducing derivatives (principle 8), catalysis (principle 9), and real time analysis for pollution prevention (principle 11). The waste impact of bioinspired silica was low, as the only wasted raw materials are water and the amine used (pentaethylenehexamine). Water has a relatively low waste severity factor of 0.5 which lowered the impact of wasting a high volume of water. On the other hand, pentaethylenehexamine has a high waste severity factor of 4.

#### Group 2. Energy Efficiency

Figure 5b shows the energy efficiency of the selected silicas. This comparison serves to distinguish the environmental costs of mesoporous silicas and shows the main barrier to large scale production. The main factor affecting these scores was the heating requirement either to maintain a reaction mixture above room temperature for several hours or for drying and purification. This is further evident when focusing on the variations between different mesoporous silicas—HMS has a very low score for group 2, COK-12 and SBA-15 show a moderate score, and MCM-41 has a very high score. These differences arise from temperatures and dwell times for synthesis and postsynthesis processing. For example, HMS synthesis occurs at room temperature with a relatively quick calcination. On the other hand, MCM-41 synthesis requires several hours at elevated temperatures followed by a longer calcination step. While SBA-15 synthesis and calcination occur at temperatures similar to those used for MCM-41 synthesis, SBA-15 synthesis is typically faster, hence it scores less than MCM-41 but higher than HMS. COK-12 synthesis on the other hand takes place at moderate temperatures, and it requires rather lengthy drying and calcination steps, both at elevated temperatures. Notably, bioinspired synthesis shows one of the lowest scores, only comparable to pyrolyzed silica. This is due to the room-temperature nature of the bioinspired process, as well as the use of acid elution for the purification of the product.

#### Group 3. Hazard Prevention

Figure 5c shows the group 3 scores for all selected silicas for comparison. Notably, a significant contribution was observed for the scores from principles 3 and 12 (safety around the raw materials and the synthesis conditions). This arises from the use of TEOS and amines, which are categorized as substances that can be toxic to the environment or human health. Further, the use of ethanolic solvents at higher temperatures also contributed to the principle 12 scores. This was particularly apparent for Stöber synthesis where the reactions are carried out in ethanolic solutions over a long duration. Solvent-intensive reactions like the Stöber synthesis and HSM also scored poorly for principle 5 (safer solvent). The similarities in scores across this group between most mesoporous and industrial materials can be attributed to the commonalities of their precursors, as well as the identically null toxicity of all the silicas produced. Significantly, a higher group 3 score for bioinspired silica was obtained, mainly from principles 3 and 12. Although bioinspired synthesis avoids organosilane precursors and alcoholic solvents, the use of amine molecules (classes as GHS category 1 irritants), contributed to the score under principle 3 (discussed further in the next section).

### Identification of Potential Improvements

In the greenness evaluation above, problematic areas were identified for some silicas. In this section, we report the results from using DOZN 2.0 in finding the combination of parameters that would result in lowering the aggregate score for selected types of silica. New set of scores were obtained for each point of testing when exploring operational parameter boundaries. The objective of this preliminary optimization shows the potential of the DOZN 2.0 tool in identifying improvements possible.

#### Mesoporous Silicas for High Value Applications: MCM-41, SBA-15, COK-12, and HMS

**Figure 6:**
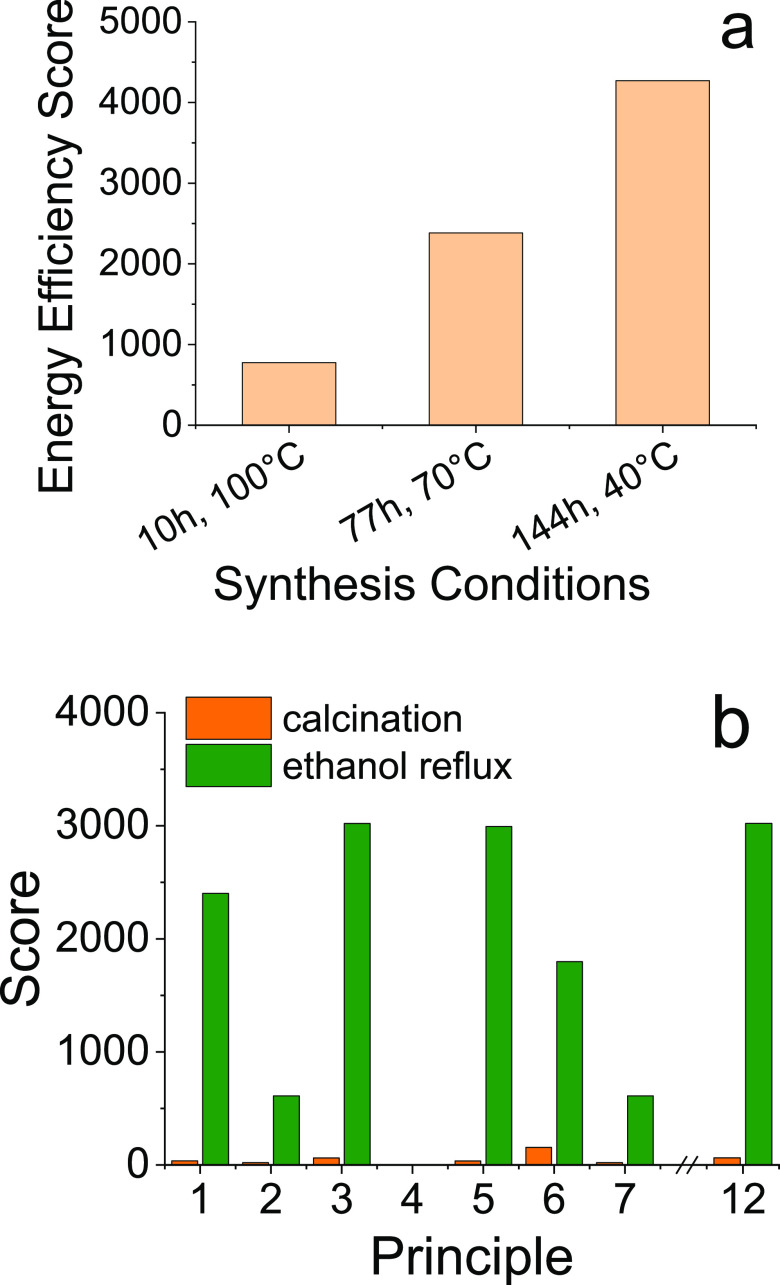
Comparative evaluation showing the effect of (a) synthesis step duration and temperature during MCM-41 formation and (b) principle scores for two purification methods used for HMS synthesis (calcination and ethanol reflux). Only those scores are included in b which presented significant variation as a result of the changes to the purification method.

The abundant literature on mesoporous silicas enables a comparison across a multitude of reaction conditions. For the present study, the reaction parameters as shown in Table 1 above were tested for their effect on greenness scores. Particularly, mesoporous silicas require long reaction times, several hours to several days, and high temperature purification processes. These conditions are mostly necessary to achieve intricate structures on the nanoscale. Namely, longer reactions are required for the formation of long-range periodic porosity around micellar nuclei, and energy-intensive purifications are required for the removal of surfactants. In order to identify the greener reaction conditions, we investigated the principle 6, energy efficiency, scores for MCM-41 as an exemplar case under various reaction times and temperatures (Figure 6a). While the synthesis literature implies that reducing the reaction temperature leads to greener synthesis of mesoporous silica, the results from DOZN 2.0 calculations show the opposite–rapid reactions at higher temperatures have higher energy efficiency (and hence a lower score). This is a very interesting result and further highlights the need for holistic evaluation in contrast to single metric (e.g., reaction temperature) approaches.^64^

A second factor influencing the 12 principle scores of mesoporous silica syntheses was found in the method used for the removal of the soft template. Given the need to free up the pores for carrier or adsorption applications, this step is highly necessary. Most commonly, this purification step is achieved by calcination in air, typically at 550 °C. The porous structures surrounding the templates result in longer calcination times to ensure complete removal (typically 5–8 h). The literature also presents an alternative purification method, which relies on using boiling ethanol for the extraction of soft templates. Note that this method offers complete removal of the template only for limited types of mesoporous silicas. This solvent reflux reduces the time and temperature required for the product purification. Figure 6b shows the comparison of purification methods for hexagonal mesoporous silica.^54^ Significantly, the increase in ethanol consumption and high energy demands for refluxing resulted in a great increase for most principle scores. This is due to an increase in mass of solvents/auxiliaries, as well as the high severity factors of ethanol, as determined by its GHS category. A potential reduction to the score could be achieved by recycling the solvent, which would eliminate the waste production and improve the atom economy of the process. However, it remains to be studied whether the downstream purification, recovery, and reuse of ethanol and the template are feasible, both from chemistry and process standpoints. Nonetheless, the use of flammable solvents at high temperatures will unavoidably result in poor scores for principles relating to accident prevention and the use of safer chemicals. This is another interesting finding, which contradicts the commonly held belief that avoiding high temperature calcinations can improve the greenness of a process.^65^

#### Bioinspired Silica

**Figure 7:**
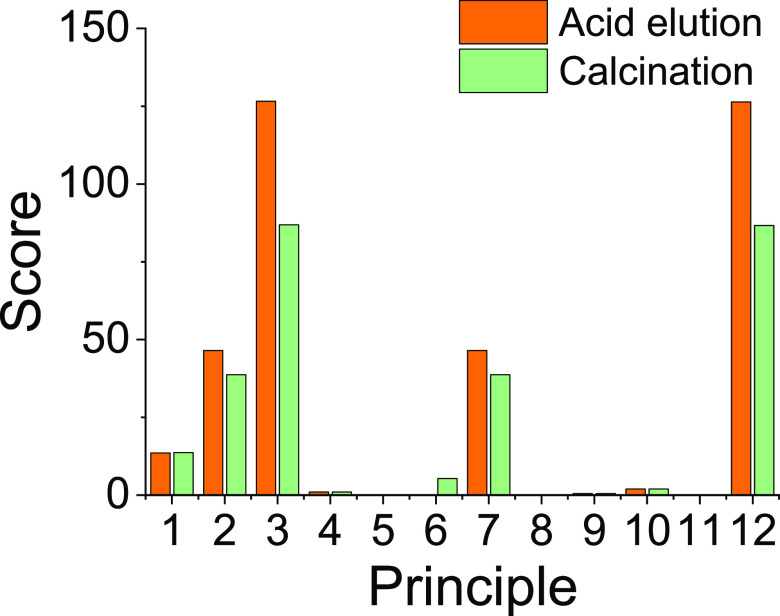
Comparison of purification methods for bioinspired silica based on their principle scores. Acid elution was carried out at pH 2 for 5 min, while calcination was performed at 550 °C for 4 h.

The DOZN 2.0 evaluation of bioinspired synthesis for high-value silica has shown a significant impact of the choice of purification method. Figure 7 shows a comparison of principle scores for pure silica obtained by calcination at 550 °C for 4 h and its comparison with a product eluted at room temperature using hydrochloric acid, as previously reported by our group.^60,66^ As expected, the use of hydrochloric acid increased the scores relating to hazard prevention and waste production, while improving the energy efficiency score. The overall score of the process remained unaffected, which is interesting, especially noting that the capital and operating costs for using calcination are likely to be higher than when using acid elution.^60^

**Figure 8:**
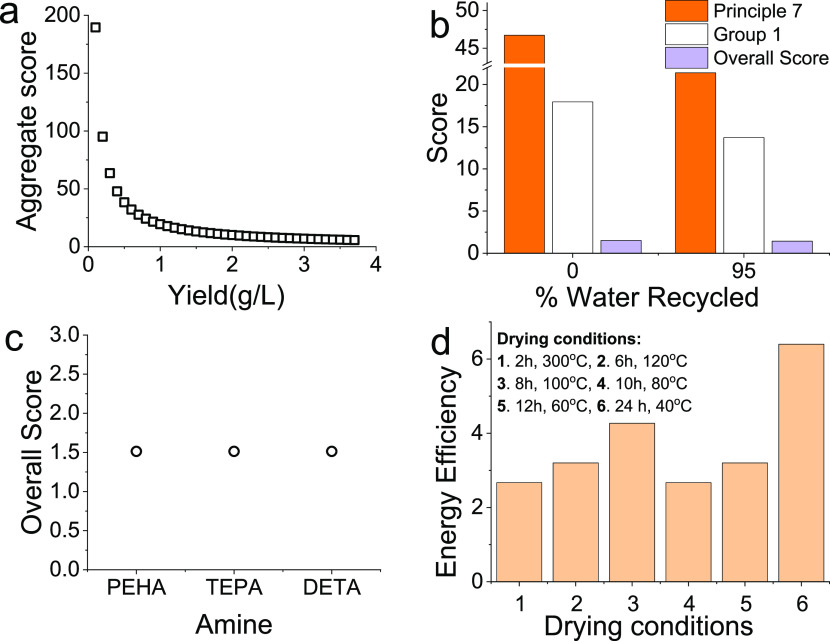
Sustainability considerations for the optimization of bioinspired silica synthesis. (a) The effect of improving yield on overall score. (b) Comparison of principle 7 (renewable feedstock), group 1 (resource efficiency), and overall score when water is recycled rather than wasted. (c) Effect of using different amines on the overall score. (d) Comparison of different drying conditions on energy efficiency for bioinspired silica.

Further, we evaluated the effect of yields (grams of silica produced per liter) of bioinspired silica on its greenness scores (Figure 8a). Here, the range of yield investigated was from 0.1 g/L (which is typical of lab-scale experiments performed at discovery stage) to 3.7 g/L (representing desired yields for industrial production). It can be seen that improving the solids yield from 0.1 g/L to 0.5 g/L significantly improved the rating (scores reducing from close to 200 to below 50). A further increase in yields showed marginal changes to the aggregate score, which suggests that high yields, which may influence the economics, are unlikely to improve the sustainability of the process. Next, we considered the effect of recycling water, which was made possible from a recent discovery.^66^ Recycling 95% of the water resulted in reducing the principle 7 score from 46 to 21 (Figure 8b). Associated reduction in group 1 (resource efficiency) was also observed (from a score of 18 to 13); however, these changes affected the overall scores negligibly (changed from 1.5 to 1.4).

As the use of certain amines in bioinspired silica synthesis was identified as a reason for poor rating in Group 3 scores, we investigated the effect of using different amines: pentaethylenehexamine (PEHA), tetraethylenepentamine (TEPA), and diethylenetriamine (DETA). Note that all these (and many more) have been reported to produce bioinspired silica.^67^ It was found that between the amines considered here, the changes to the overall score were insignificant (Figure 8c).

Finally, as the synthesis of the bioinspired silica occurs at room temperature, the only step affecting the energy efficiency score was the drying stage. In order to explore the impact of drying on the scores, a range of different times and temperature were evaluated (Figure 8d). Both 2 h at 300 °C and 10 h at 80 °C achieved identical lowest scores. These two different conditions however will have an effect on the properties of the silica, so it is important to understand this when choosing the time and temperature and to strike the right balance between greenness and product quality.

An important consideration of the process comparisons shown in Figures 7 and 8 is the effect that such changes to a parameter can have on the properties of the silica product and their performance. Therefore, with selected examples, we discuss the impact of changes in process conditions on the materials properties. Figure 7 shows the effect that room temperature acid elution has on the different principles when used as an alternative to calcination at higher temperatures. The room-temperature acid elution is as effective as calcination in removing the amine.^60^ Further, the mild nature of acid elution also avoids the degradation of porous structures, resulting in a higher surface area when compared to calcination. Starting with higher concentrations of silicate precursor, which leads to a greener process (Figure 8a), in fact led to a more complete condensation of silica, and more efficient coagulation, without any significant impact on the materials properties.^68^ It is well-documented that the additive structure plays a crucial role in controlling the process and silica properties; the amine chain lengths, architectures and their protonation behaviors have all been shown to affect the formation and properties of silica.^20,67,69^ It is therefore interesting to note that the variation in organic additive structure shows that the sustainability of the process remains largely unchanged (Figure 8c), and hence the properties and structures of silica can be tuned by using different amines, yet without affecting the greenness scores. In future studies, it would be interesting to explore a much wider range of amines reported for BIS synthesis in order to understand their effects on the greenness scores as well as the quality of silica produced.

## Discussion and Conclusions

Silica has proven to be an interesting case study for the validation of the DOZN 2.0 green chemistry evaluator. By comparing significantly different processes, which produce chemically identical materials with largely identical toxicities, DOZN 2.0 has been shown to provide users with a thorough understanding of the environmental consequences of synthesis and processing. More importantly, the ability to evaluate any chemical process in a rapid fashion constitutes an asset for implementing sustainability into the design of new processes and materials. The flexibility of implementation means that DOZN 2.0 can act as an ideal exploratory or indicative tool for surveying a myriad of processes or materials. Once a process or product has been shown to be greener than its alternatives, then a more thorough and resource-intensive evaluation can be undertaken, such as lifecycle assessments.

The findings shown here are consistent with previous evaluations of sustainability of silica technologies found in the literature.^70,71^ Namely, the energy-intensive reaction conditions and subsequent removal of soft templates makes most sol–gel silica processes unsustainable. However, DOZN 2.0 has enabled the quantification of these limitations for the first time.

By tracing the overall score of a product to the individual contributions of each principle score, it was possible to determine which elements of green chemistry posed a significant challenge for each of the processes. For the highest scoring silicas, the main green chemistry principles resulting in poor sustainability scores were generally found to be inherently safer chemistry for accident prevention (principle 12), less hazardous synthesis (principle 3), safer solvents and auxiliaries (principle 5), design for energy efficiency (principle 6), waste prevention (principle 1), atom economy (principle 2), and use of renewable feedstocks (principle 7). Bearing in mind the importance of the remaining 5 principles, it is valuable to identify the main areas of opportunity that result in the highest environmental cost for most high-value silicas.

Each of these challenges requires a different solution, several of which can only be implemented by redesigning processes and materials starting from a sustainability perspective. For instance, the need for harsh chemicals and reaction conditions stem from the mechanism of hydrolysis of tetraethyl orthosilicate (TEOS) as the first step in silica formation. Therefore, a process can be inherently greener when it avoids the use of TEOS. Likewise, the use of solvents and hazardous chemicals at higher temperatures increases the risk of accidents during manufacturing and is therefore detrimental to sustainability as per principle 12. This challenge can be avoided by taking advantage of the mechanistic phenomena that underpin the sol–gel formation of silica nanomaterials.^19^ Using this knowledge, it is possible to overcome the need for energy intensive and harsh reaction conditions. Once a reaction mechanism has been identified, which minimizes energy requirements and hazardous conditions, the reaction can be optimized by standardizing the process. Maximizing yield while minimizing waste are not only central improvements toward a sustainable process but also necessary engineering efforts to improve the capital expenditure (CAPEX) of production and the operational expenditure (OPEX) of waste management. Nonetheless, other potentially impactful solutions can be retrofitted into existing infrastructures, as is the case with principle 7, the use of renewable feedstocks and recycling solvents.

When surveying the different types of silicas herein, it becomes clear that while low value silicas have excellent greenness scores, high-value silicas perform poorly on this scale. This highlights the tension between high-value silicas that are desired for emerging markets and the sustainability of their synthesis. The present comparison has shown bioinspired silica to be a promising alternative to conventional high-value silicas while providing excellent sustainability. The suitability of bioinspired silica for high-value applications such as drug delivery, catalysis, and water remediation has been previously evaluated,^72^ showing comparable performance to MCM-41. The industrial achievability of nanomaterials can be described as dependent on three major factors: scalability of their synthesis, their economic feasibility, and their sustainability. As discussed above, bioinspired synthesis has been proven to produce high-value porous and functional silicas using significantly lower energy inputs than the conventional routes to such high-value materials. In addition, it eliminates the need for hazardous operational conditions. These two factors alone have a major impact on the scalability and economic feasibility of bioinspired synthesis, as previously shown by techno-economic analysis and scale-up studies using batch and continuous processes.^20,73^

The evaluation also showed the importance of avoiding solvents during processes and particularly as waste. As an example, Stöber synthesis was shown to be one of the most hazardous processes in our evaluation due to the extensive use of ethanol at high temperatures. On the other hand, bioinspired silica showed a perfect score in principle 5 due to all steps of the process being carried out only using water as a solvent, which inputs a hazard score of 0 to the DOZN 2.0 algorithm.

Another important limitation to the manufacturing of nanomaterials is the higher production of waste than that of their bulk counterparts. Previous studies have used E-factor analysis (waste-to-product ratio) and concluded that conventional nanomaterial synthesis produces up to 1000 times more waste than the production of bulk materials.^74^ These findings are consistent with the results calculated by DOZN 2.0, which show principle 1 scores for waste reduction to be 3 to 30 times higher for lab-based silica processes than for industrialized materials. The only two exceptions to this trend were SBA-15 and bioinspired silica. Of these two, SBA-15 produces ethanol and CO_2_ as waste from the conversion of TEOS and the calcination of pluronic surfactant, respectively. On the other hand, bioinspired silica produces NaCl as a waste from the neutralization of sodium metasilicate using HCl. Therefore, while the proportion of waste produced from both processes is comparable to that of current industrial products, only bioinspired silica produced nonhazardous waste that can be managed in an economical and sustainable manner. Such crucial considerations are exempt from the E-factor analysis, which only takes into consideration the amounts of waste and product and not the toxicity of the waste. Finally, bioinspired silica has the unique advantage of producing high-value functional nanosilica at room temperature; its implementation would also be safe and inexpensive.^20,72^

## Supporting Information Available

A table containing product information, reaction conditions, raw materials, and process information (XLSX)
